# GINS2 Promotes Osteosarcoma Tumorigenesis via STAT3/MYC Axis

**DOI:** 10.1155/2023/8454142

**Published:** 2023-02-23

**Authors:** Bingkai Ren, Yibin Zheng, Lizhong Nie, Fanhui Wu, Leiwen Huang, Jingdu Wei, Dong Yang

**Affiliations:** ^1^Department of Orthopedics, The First Affiliated Hospital of Nanchang University, Nanchang, China; ^2^Department of Orthopedics, The Yiwu Central Hospital, Jinhua, Zhejiang, China

## Abstract

GINS2 is overexpressed in several cancers, but little is known about its role in osteosarcoma (OS). A series of in vivo and in vitro experiments were conducted to explore the role of GINS2 in OS. In this study, we demonstrated that GINS2 was found to be highly expressed in OS tissues and cell lines, which was associated with poor outcomes in OS patients. GINS2 knockdown hindered the growth and induced apoptosis in OS cell lines in vitro. Furthermore, GINS2 knockdown effectively inhibited the growth of a xenograft tumor in vivo. By using an Affymetrix gene chip and intelligent pathway analysis, it was demonstrated that the GINS2 knockdown could reduce the expression of several targeted genes and reduce the activity of the MYC signaling pathway. Mechanically, LC-MS, CoIP, and rescue experiments revealed that GINS2 promoted tumor progression through the STAT3/MYC axis in the OS. Moreover, GINS2 was associated with tumor immunity and may be a potential immunotherapeutic target for OS.

## 1. Introduction

Osteosarcoma (OS) is a type of primary malignant bone cancer that occurs with a high frequency, accounting for 60% of cases in adolescents [1, 2]. The overall incidence of OS in the United States was approximately 4.5 per million [3]. To inhibit tumor growth and metastasis, chemotherapy, and radiotherapy are the main treatment options for OS [4]. Therefore, surgery combined with chemotherapy is the main treatment for osteosarcoma. However, most OS will eventually acquire resistance to these therapies. Moreover, osteosarcoma patients had poor prognoses when occurred with distant metastases. It means the 5-year overall survival rate is less than 25% [5, 6]. There are still no effective therapies for metastatic osteosarcoma. However, the underlying molecular mechanisms of osteosarcoma progression are not fully elucidated.

GINS is a eukaryotic replication helicase that consists of four subunits including Sld5, Psf3, Psf2, and Psf1 which play a central role in the cell cycle and DNA replication [7, 8]. GINS2, also known as Psf2, is a member of the GINS complex. GINS2 was overexpressed in multiple tumors and reported to be involved in tumorigenesis in several types of cancers including breast cancer [9], leukemia [10, 11], and lung cancer [12, 13]. For example, GINS2 knockdown inhibits cell proliferation and induces apoptosis in a human lung adenocarcinoma cell line and pancreatic cancer cell lines. All these findings suggest that GINS2 is involved in the progression of multiple cancers. However, the roles of GINS2 in OS progression remain unknown.

In this study, we first showed that GINS2 was an oncogene and highly correlated with cancer progression as well as patient survival prognosis. The data from bioinformatics, in vitro, and in vivo studies demonstrated that GINS2 promoted cellular malignancy and may be a potential immunotherapeutic target for OS. Mechanistically, GINS2 activated the MYC pathway and ultimately induces carcinogenicity by upregulating STAT3. Thus, these findings imply GINS2 promoted tumor progression through the STAT3/MYC axis in the OS. It provides new insights into the development of new treatment strategies using GINS2 as a therapeutic target for the clinical treatment of OS.

## 2. Materials and Methods

### 2.1. Patient Samples and Follow-up

We selected 59 cases of OS from the databases of the First Affiliated Hospital of Nanchang University, China, and obtained their corresponding tissue samples. Before the biopsy stage, the patients had not received radiotherapy and preoperative chemotherapy. When OS was diagnosed, all patients received combined chemotherapy, such as high-dose ifosfamide and doxorubicin, cisplatin, and methotrexate, and then underwent a partial removal of the primary tumor, which was followed by an adjuvant chemotherapy cycle. After the completion of chemotherapy, patients were followed up every 3 months for 6 years.

### 2.2. Immunohistochemical Staining for GINS2 in OS Samples

The endogenous peroxidase was inactivated in the paraffin-embedded tissue sections by the treatment with hydrogen peroxide. The antigen was recovered by adding 10 mmol/L citrate buffer at pH 6.0 to the sections and microwaving them. Next, the sections were incubated with the anti-GINS2 antibody (Sigma) at 4°C overnight, and protein expression was detected using a secondary antibody (Sigma). During each immunohistochemical test, negative and positive controls were included. The immunohistochemical staining was systematically and comprehensively assessed by two independent pathologists, and the patient's identity was kept confidential. The intensity and positive staining rate (0/1+/2+/3+) in the cytoplasm and cell membranes of the cancer tissue and adjacent tissue (epithelial) samples were evaluated separately. Samples were grouped based on the product of the “staining intensity score” and the “staining positive rate score” as the total score, and samples with a total score ≤6 were defined as the antibody low expression group and those with a score >6 were designated as the antibody high expression group.

### 2.3. Cells Lines and Cell Culture Conditions

OS cell lines were obtained from our laboratory. U2OS and HOS cell lines were kept in DMEM, and Saos-2 cells were kept in McCoy's 5A medium. The medium was supplemented with 10% fetal bovine serum (FBS; Gibco). The cells were cultured in sterile conditions in a humidified incubator at 37°C and 5% CO_2_ in the air, and the medium was replaced every 2 days. When the cells reached 75% confluence, they were harvested using trypsin for *in vivo* and *in vitro* experiments.

### 2.4. Quantitative Real-time Reverse Transcriptase-Polymerase Chain Reaction

A total RNA was extracted using TRIzol (Superfec TRI, Shanghai, China), and RT-qPCR was performed using a Promega M-MLV RT-qPCR Kit. The expression of GINS2 was quantified using M-MLV-R Tase (Promega), and GAPDH was used as an internal control. The primers used for real-time RT-qPCR were as follows: GINS2: forward primer: CAGAAATGTCGCCTGCTCC, reverse primer: GGATTTCGTCTGCCTTCG. GAPDH: forward primer: TGACTTCAACAGCGACACCCA. Reverse primer: CACCCTGTTGCTGTAG CCAAA. Relative expression levels were calculated using the comparative △Ct method (△CT values = Ct value of target gene − Ct value of reference gene). △CT values less than 12 were considered high abundance expression, 12–16 as medium abundance expression, and greater than 16 as low abundance expression.

### 2.5. Lentivirus-Vector Construction and Cell Transfection

The *GINS2* gene was used as a template to construct a lentiviral vector (LVpGCSIL-004PSC24135-1) for shRNA interference. The infectious shRNA lentivirus was delivered into 293T cells, purified, and used to infect HOS, Saos-2, and U2OS cells to obtain stable clones. The fluorescence was measured 72 hours after infection in an inverted fluorescence microscope to evaluate the infection efficiency. When the infection rate has reached 80%, RT-qPCR is used to measure the expression level of *GINS2*.

### 2.6. Cell Proliferation Analysis

In short, OS cells infected with shRNA lentivirus or shGINS2-lentivirus control were trypsinized (Sangon Biotech) in the logarithmic growth phase, resuspended in a standard medium, and then seeded into a 96-well (2 × 10^3^ cells/well) plate. After the plating was completed, at room temperature, the plates were scanned under the green fluorescence every day for 5 consecutive days, and a Celigo®Image Cytometer (Nexcelom) was used to evaluate the number of cells.

In the rescue experiment, we utilized the MTT method to evaluate cell proliferation. The cells were seeded on 96-well plates at a density of 2 × 103 cells/mL in the STAT3, shGINS2, and shCtrl overexpression groups. The time period was 1–5 days, and each group was seeded in three wells for cultivation. After the incubation was completed, we added 10 *μ*L of 5 mg/mL MTT to each well and incubated the cells at 37°C for 4 hours. Then we removed the medium and added 150 *μ*L of dimethyl sulfoxide (DMSO). We used an enzyme-linked immunosorbent assay (ELISA) reader to measure the absorbance at 490 nm. We used the absorbance values to draw the cell proliferation curve.

### 2.7. Cell Apoptosis Analysis

Annexin V-APC was used for staining, and then flow cytometry was used to effectively evaluate cell apoptosis. The cells were placed at 37°C for 48 hours. After that, the cells were centrifuged at 100 × g, washed twice with PBS, and resuspended in 1 × binding buffer. The pellet was resuspended in Annexin V-APC and propidium iodide, incubated at room temperature in the dark for 15 minutes, and washed twice with PBS. The analysis was based on the FACS Calibur flow cytometer (BD Biosciences). All experiments were performed three times.

The Caspase-3/7 Assay Kit (Promega) was used to detect the activity of caspases 3/7 in the OS cells according to the manufacturer's instructions. The fluorescence intensity in the cells was quantitatively evaluated at the excitation of 499 nm using an ELISA tablet counter.

### 2.8. Cell Cycle Distribution

To assess cell cycle distribution, U-2OS cells were trypsinized, resuspended in 70% ethanol, and incubated overnight at 4°C. The fixed cells were centrifuged, washed in ice-cold PBS, and then incubated in RNase A at 37°C for 30 min and in 400 *μ*L of propidium iodide (BestBio) at 4°C for 30 min. Annexin V/propidium iodide (PI) apoptosis detection kit (Sigma) was used to assess the cell cycle according to the manufacturer's instructions. A FACS Calibur flow cytometer (BD Biosciences, San Jose, USA) and ModFit 3.0 software (Verity Software House, Topsham, USA) were used for analysis.

### 2.9. Transwell Assay

The cells were resuspended in 100 *μ*L of serum-free medium and then inoculated into the 8 *μ*m chamber coated with Matrigel. The chamber was incubated in 500 *μ*L of complete medium for 24 hours. We use a cotton swab to remove the remaining Matrigel and stain the cells in the upper chamber. The chamber was immersed in 4% paraformaldehyde (PFA), and then the cells were stained with crystal violet dye. Six microscope fields were imaged, and the number of cells in these fields was counted (100× magnification).

### 2.10. Wound Healing Assay

For this assay, 5 × 10^4^ cells were plated in 6-well plates and grown until they reached 90% confluency. We used a 10 *μ*L plastic pipette tip to make a scratch. Wound healing images were taken at 0, 4, and 8 hours. At the same time, we calculated the cell migration distance and compared it with *T* = 0 hours.

### 2.11. In Vivo Xenograft Mouse Model

Animal care procedures and mouse experiments have been approved by the relevant units. Experiments were performed in female Fox Chase severe combined immunodeficiency 6-week-old mice. We split mice into control and experimental groups. HOS cells stably expressing shGINS2 or GINS2 were suspended in PBS (4 × 10^6^ cells in 200 *μ*L) and inoculated subcutaneously in the right axillary position (*n* = 10 per group). Data collection began 12 days after the mice were subcutaneously injected with OS cells (weigh the nude mice and measure the length and short diameter of the tumor) and then collected once every 2 days for a total of 5 times. After 4 weeks, the mice were sacrificed, and their internal tumors were analyzed.

### 2.12. Microarray Gene Expression Analysis

After the infection with shGINS2, a total of RNA was extracted from HOS cells. We used the NanoDrop 2000 spectrophotometer and Agilent 2100 bioanalyzer system to evaluate the concentration and quality of RNA. Qualified samples were analyzed using the GeneChip PrimeView human gene expression array (Affymetrix, USA). The analysis results were selected according to the critical *P* value <0.05 for the selection of differentially expressed genes. At this time, the critical value of the fold change was equal to 2. We used “subtle path analysis” to analyze the differentially expressed genes obtained in microarray analysis. Specifically, we evaluated the molecular pathways and core organisms in which the differentially expressed genes were involved.

### 2.13. Western-blot

Total protein was extracted using a lysis buffer and protease inhibitor (Beyotime Biotechnology). Equivalent protein amounts were denatured in an SDS sample buffer and then separated by SDS-PAGE and transferred onto a polyvinylidene difluoride membrane. After being blocked with 5% nonfat dry milk in TBST, the blotted membranes were incubated with anti-GINS2 antibodies (1 : 500, SIGMA) and then incubated with a secondary antibody (1 : 2000, Santa Cruz, USA). GAPDH protein levels were also determined by using the specific antibody (1 : 2000, Santa Cruz, USA) as a loading control.

### 2.14. LC-MS Analysis and Coimmunoprecipitation

The fusion *GINS2* gene was amplified by adding a 3 × FLAG tag sequence (Sigma) using PCR and inserted into the GV208 lentiviral vector. The construct is called 3 × FLAG-GINS2. Lentiviral empty vector and 3 × FLAG-GINS2 plasmid were cotransfected along with the helper plasmid into 293T cells, and the lentiviral particles (lenti-control (NC) and lenti-3 × FLAG-GINS2 (OE)) were harvested. We lysed the NC and OE stable cell lines based on the radioimmunoprecipitation assay, and the total protein in the supernatants was collected by centrifugation to quantify proteins using the bicinchoninic acid assay (BCA). Equal amounts of total protein from the two groups were used for coimmunoprecipitation (Co-IP) with FLAGbeads (Sigma), and the subsequent in-depth SDS-PAGE and Coomassie brilliant blue staining. Proteins in the gel bands were further hydrolyzed using trypsin to generate peptides for liquid chromatography−mass spectrometry (LC−MS) identification. The protein identification results in each sample were obtained by performing a database search using the PD/MASCOT software. Finally, bioinformatics analysis of the proteins specifically identified in the OE group was performed, and the gene network map was drawn.

### 2.15. Prediction of Immunotherapy Response

The Tumor Immune Dysfunction and Exclusion (TIDE) algorithm is a computational mechanism that uses gene expression profiles to forecast immune checkpoint inhibitor responses. We collected gene expression profiles of 47 osteosarcoma samples from the GSE39058 cohort. And TIDE algorithm was used to predict the immunotherapy response of patients with osteosarcoma. The deconvolution consequences for the tumor-infiltrating immune cells were analyzed through the CIBERSORT algorithm.

### 2.16. Statistical Analysis

Three experiments were performed, and the data was calculated according to the mean ± standard deviation (SD), and the analysis was performed with the help of SPSS 23.0 and GraphPad Prism 7.0. We used the Student's *t*-test and analysis of variance (ANOVA) to assess the differences between the two groups. *P* < 0.05 was used to indicate statistical significance.

## 3. Results

### 3.1. GINS2 Level Correlates with Tumor Progression and Predicts Clinical Outcomes of OS Patients

**Figure 1:**
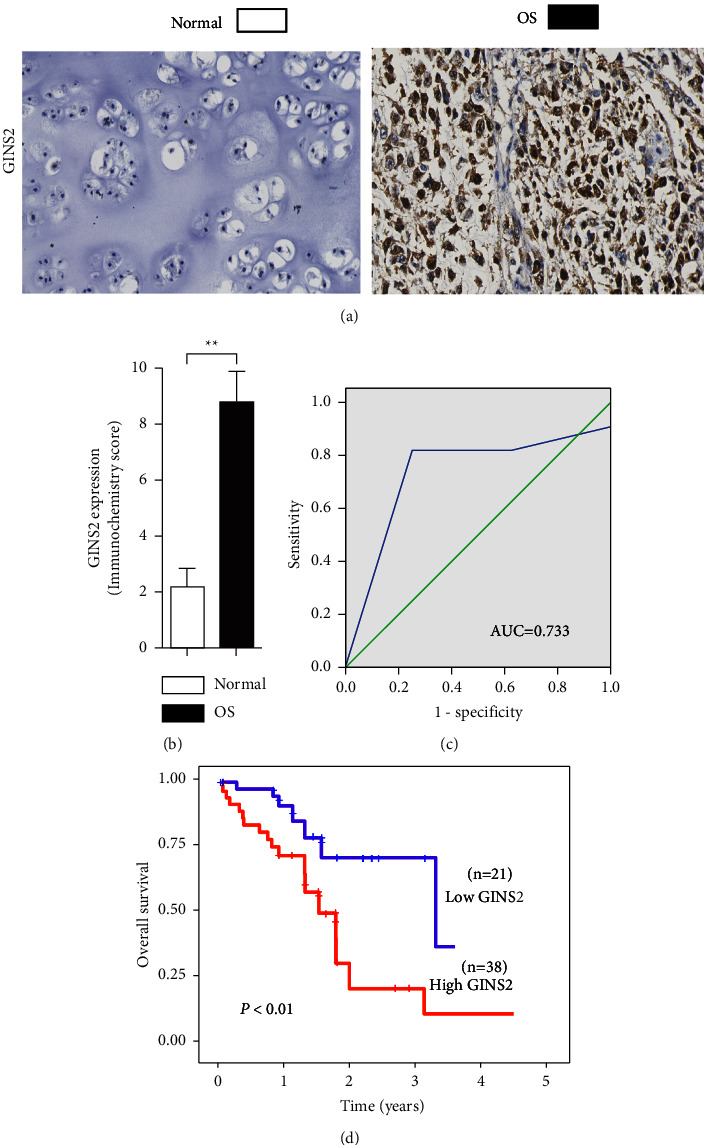
GINS2 is overexpressed in OS patients and associated with poor survival. (a) Immunohistochemical analysis of the expression of GINS2 in OS and normal tissues. (b) The immunohistochemistry score of GINS2 in OS (filled bar) and normal tissues (open bar) tissues were plotted. (c) The ROC curve was executed to identify the cutoff value for GINS2. (d) Kaplan–Meier analysis for overall survival with low or high expression of GINS2. All data were presented as the mean ± SD, statistical significance was analyzed by Student's *t*-test and one-way ANOVA, ^*∗*^*P* < 0.05, ^*∗∗*^*P* < 0.01.

To systemically study the role of GINS2 in osteosarcoma (OS), we analyzed the protein levels of GINS2 in 59 OS tissues with 8 normal tissues by immunohistochemistry (IHC) and found that the protein levels of GINS2 are significantly elevated in OS tissues (*P*  <  0.01, Figures 1(a) and 1(b)). According to the receiver operating characteristic (ROC) curve (Figure 1(c)) with an optimal cutoff point of 6.0, we found that GINS2 is overexpressed in 64.4% (38 of 59) of human OS specimens (Table 1). To determine the clinical importance of GINS2 in human OS, correlation analysis revealed that elevated GINS2 isn't correlated with the characteristics of OS patients (*P*  >  0.05) (Table 1). However, Kaplan-Meier analysis showed that patients with a higher level of GINS2 were associated with overall survival (*P*  <  0.01, Figure 1(d)). Together, these data demonstrated that GINS2 was a prognostic marker, suggesting that GINS2 might possess a promoting role in OS malignancy.

### 3.2. GINS2 Promotes OS Cell Growth and Metastasis

**Figure 2:**
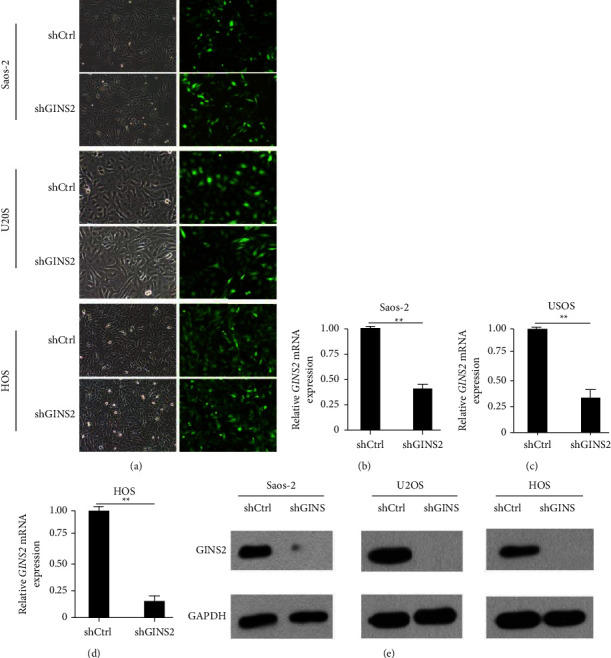
Lentivirus-mediated GINS2 knockdown specifically inhibited GINS2 expression in OS cells. (a) Saos-2, HOS, and U2OS infected with GINS2 -shRNA or shCtrl lentivirus and nontransfected cells were examined by light microscopy and fluorescent microscopy 3 days after infection (100×). More than 80% of cells expressed GFP. (b–e) Efficient knockdown of GINS2 in OS cell lines. Saos-2 (b), U2OS (c), and HOS (d) cells were transduced with lentivirus expressing control or GINS2 shRNA. The knockdown efficiencies were detected with RT-qPCR and WB. All data were presented as the mean ± SD, statistical significance was analyzed by Student's *t*-test and one-way ANOVA, ^*∗*^*P* < 0.05, ^*∗∗*^*P*  <  0.01.

**Figure 3:**
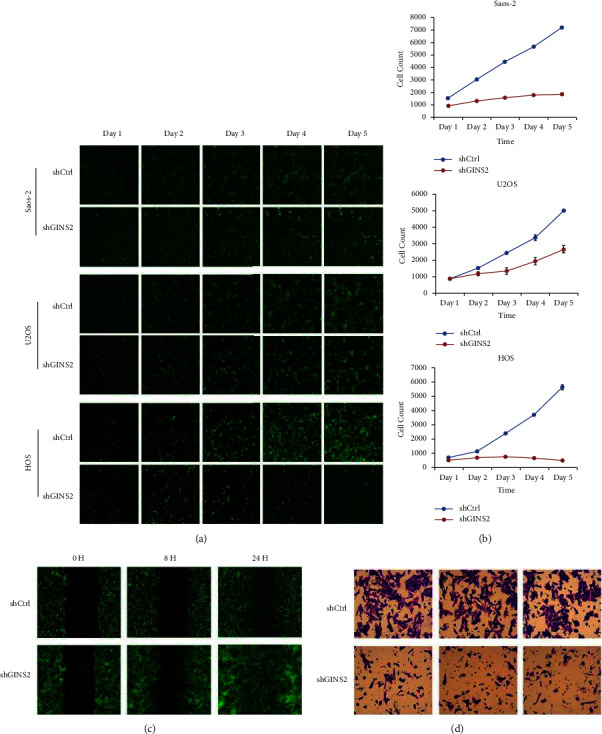
Knockdown of GINS2 inhibited cell proliferation and migration. (a) Representative images of OS cells with or without GINS2-depletion continuously for 5 days. (b) HCS assay of cell proliferation of Saos-2, U2OS, and HOS cells. (c-d) Wound-healing and migration analysis for the effect of GINS2 gene reduction on the HOS cell migration. All data were presented as the mean ± SD, statistical significance was analyzed by Student's *t*-test and one-way ANOVA, ^*∗*^*P* < 0.05, ^*∗∗*^*P*  <  0.01.

In accordance with GINS2 expression in OS patients, the RT-qPCR assay demonstrated that GINS2 was highly expressed in the three OS cell lines. To gain insights into the potential role of GINS2 in OS progression, we constructed stable GINS2 knockdown (shGINS2) cells using Saos-2, U2OS, and HOS, respectively (Figures 2(a)–2(e)). Cell proliferation monitored by high-content screening (HCS) analysis showed that depletion of GINS2 obviously inhibited cell proliferation in all three OS cells (Figures 3(a) and 3(b)). To confirm the relationship between GINS2 and cell metastasis, a transwell assay and wound healing analysis were performed. The results showed that GINS2 silencing markedly reduced the migration and invasion of OS cells (Figures 3(c) and 3(d)). These observations are in line with the fact that elevated levels of GINS2 in OS were accompanied by aggressive progression and poor outcomes.

### 3.3. Knockdown of GINS2 Promotes Apoptosis and Induces Cell Cycle Arrest

**Figure 4:**
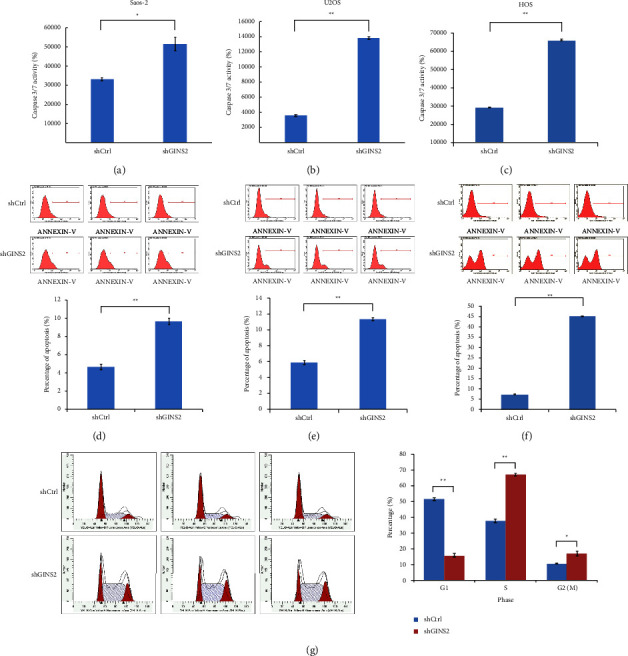
Knockdown of GINS2 promoted apoptosis in OS cells: (a–c) Activation of caspase 3/7 was further increased in GINS2-shRNA-treated OS cells compared with the control group in Saos-2 (a), U2OS (b), and HOS (c) cells; (d–f) apoptosis was determined by FACS in three OS cell lines with GINS2 knockdown and control cells. The apoptotic rate was calculated as the percentage of annexin FITC positive; (g) the cell cycle of USO2-shGINS2 cells was monitored by FACS analysis. All data were presented as the mean ± SD, statistical significance was analyzed by Student's *t*-test and one-way ANOVA, ^*∗*^*P* < 0.05, ^*∗∗*^*P*  <  0.01.

Subsequently, we evaluated the caspase-3/7 activity in GINS2 knockdown cells (Saos-2, U2OS, and HOS). Compared to the control group, caspase 3/7 activity was significantly increased in the GINS2 knockdown cells (Figures 4(a)–4(c)). In addition, Annexin V-APC staining was evaluated to analyze cell apoptosis by flow cytometry assays. The results suggested that silenced GINS2 promoted cell apoptosis (Figures 4(d)–4(f)). In order to further analyze the effect of GINS2 on cellular behaviors, a flow cytometry assay was used to monitor cell cycle phases in U2OS cells. It showed that the percentage of cells in the S phase and G2/M phase was significantly increased, while the percentage of cells in the G1 phase was reduced in the GINS2 knockdown group. It suggested that the cell cycle was blocked in the S and G2/M phases (*P*  <  0.05, Figure 4(g)). These data support the notion that GINS2 may be involved in the regulation of OS progression.

### 3.4. Silencing of GINS2 Inhibits Tumourigenicity In Vivo

**Figure 5:**
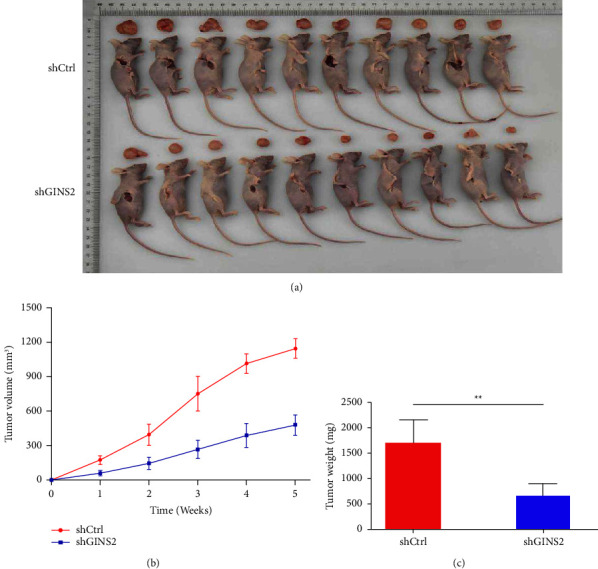
Silencing GINS2 inhibitors tumor growth in vivo: (a) xenografted tumors from nude mice were harvested at the end of the experiments; (b-c) Growth curves of tumor growth of the U2OS cells stably expressing shGINS2 (b) and weight (c) at the end of the experiments. All data were presented as the mean ± SD, statistical significance was analyzed by Student's *t*-test and one-way ANOVA, ^*∗*^*P* < 0.05, ^*∗∗*^*P*  <  0.01.

To investigate the role of GINS2 on tumorigenicity in vivo, we inoculated U2OS-shCtrl and U2OS-shGINS2 cells into the right flanks of nude mice. The animals were sacrificed at the end of the experiment, and the tumors were dissected and weighed. The tumors derived from the cells with GINS2-depleted tumors were smaller and lighter (Figures 5(a)–5(c)). This was consistent with the result of our in vitro experiments demonstrating that GINS2 knockdown impaired the proliferation of OS cells.

### 3.5. GINS2 Promotes Tumor Progression via the MYC Pathway

**Figure 6:**
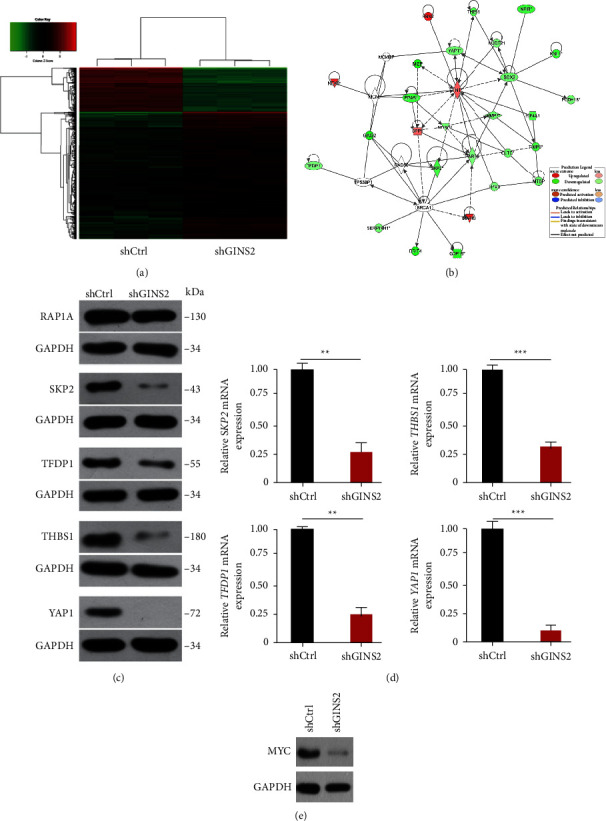
GINS2 regulates downstream molecular signaling pathways: (a) heatmap representation of genes significant differential expressions in U2OS cells infected with lentivirus expressing shCtrl or shGINS2 under the criteria *P*  <  0.05 and |fold change| > 1.5 (green represents downregulated genes while red represents upregulated genes); (b) the IPA was analyzed to complete the identification of the related molecular networks; (c-d) Western blot and RT-qPCR to verify the expression of target genes on the MYC signaling pathway; (e) inhibit GINS2 on HOS cells to detect MYC expression. All data were presented as the mean ± SD, statistical significance was analyzed by Student's *t*-test and one-way ANOVA, ^*∗*^*P* < 0.05, ^*∗∗*^*P*  <  0.01.

Since GINS2 has been reported to be involved in multiple signaling pathways in a wide spectrum of cancer types, the core mechanism of GINS2-mediated tumor progression is still unclear. We decided to conduct Affymetrix gene chips and Intelligent Pathway Analysis (IPA) to investigate the role of GINS2 in U2OS cells (Figure 6(a)). A comparison with shCtrl cells revealed that 265 genes were up-regulated and 755 genes were down-regulated in GINS2 knockdown cells. IPA revealed that multiple signaling pathways related to cancer development and apoptosis, such as the mTOR and IL-8 signaling pathways, were inhibited by GINS2. In addition, the gene list obtained from the microarray analysis was uploaded to the IPA system, and the key biological pathways were analyzed and processed to complete the identification of the related molecular networks. It was found that the expression of target genes in the MYC pathway including *YAP1, SKP2, TFDP1,* and *THBS1* was significantly suppressed by GINS2 knockdown (Figure 6(b)). These results were confirmed by RT-qPCR and western blot (Figures 6(c) and 6(d)). In order to further examine the potential mechanisms between GINS2 and MYC, we conducted RT-qPCR and Western blot assays, which showed that MYC was significantly down-regulated by GINS2 depletion (Figure 6(e)). Together, the mechanism of GINS2-mediated tumorigenicity may be involved in the MYC signaling pathway.

### 3.6. STAT3 is Required for GINS2-Mediated OS Malignancy

**Figure 7:**
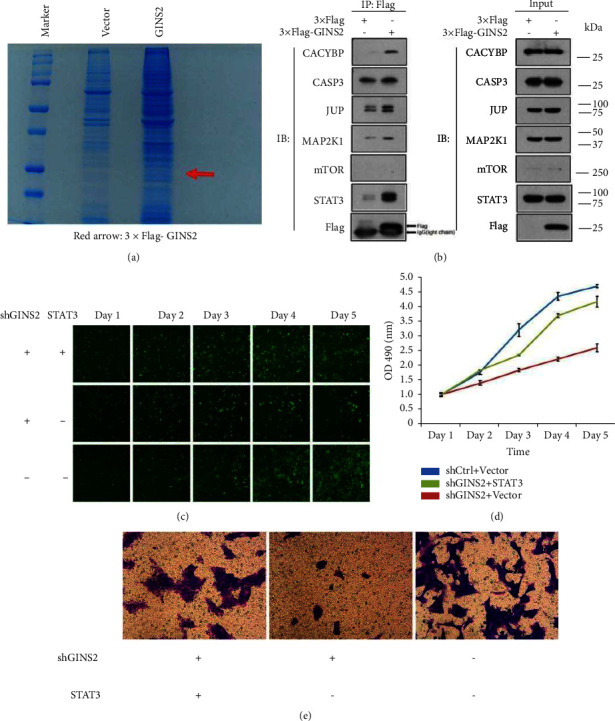
Co-IP and LC-MS identified the downstream interacting protein STAT3 and rescue experiments indicated that overexpressed STAT3 can restore the effect caused by knocking out GINS2. (a) The 3 × Flag-GINS2 interacting complex was purified using the anti-FLAG magnetic beads (red arrow: 3 × FLAG-GINS2). (b) Interaction between GINS2 and STAT3 was confirmed in HOS cells by Co-IP/immunoblotting. (c, d) HCS analysis of the effect of overexpression of STAT3 on the proliferation of HOS cells. (e) The Transwell experiment analyzes the effect of overexpression of STAT3 on HOS cell migration. All data were presented as the mean ± SD, statistical significance was analyzed by Student's *t*-test and one-way ANOVA, ^*∗*^*P* < 0.05, ^*∗∗*^*P*  <  0.01.

To determine the role of GINS2 in tumorigenicity, we used SDS-PAGE to separate the coimmunoprecipitated proteins and stain them (Figure 7(a)). The Coomassie brilliant blue staining of the gel with GINS2-IP identified several bands that were not present in the vector or IgG controls. The immunoprecipitated protein was extracted from the gel, digested by trypsin, and then subjected to the LC-MS analysis. Following coimmunoprecipitation and western blot, we identified STAT3, a core MYC interactor that existed as a candidate interaction partner of GINS2 (Figure 7(b)). We further verified the interaction between GINS2 and STAT3 in functional experiments in vitro. STAT3 was overexpressed in the GINS2 knockdown cell lines by lentivirus infection. A series of experiments showed that STAT3 overexpression restored cell proliferation and metastatic capacity in GINS2 knockdown cells (Figures 7(c)–7(e)).

### 3.7. Role of GINS2 in Immunotherapy Response

**Figure 8:**
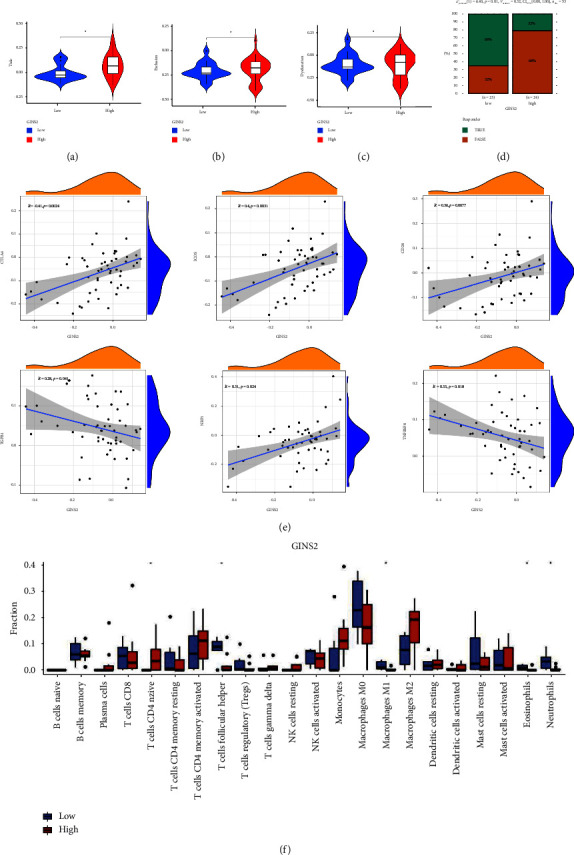
Role of GINS2 in immunotherapy response: (a–c) TIDE scores, dysfunction scores, and exclusion scores in different GINS2 groups by GEO dataset GSE39058; (d) the relationship of GINS2 and immunotherapy response; (e) the correlation between GINS2 and 6 immune checkpoints; (f) the infiltrating levels of 22 immune cell types in high/low GINS2 groups in the OS.

Given that patients with a higher tumor immune dysfunction and exclusion (TIDE) score have a higher chance of immune escape, they have a lower response rate to immunotherapy. The TIDE algorithm result of GSE39058 showed that TIDE scores and exclusion scores were lower in the low GINS2 group (*P*  <  0.05, Figures 8(a)–8(c)). It indicated that higher GINS2 did have a lower rate of immunotherapy response (Figure 8(d)). Moreover, immune checkpoints are important predictors of immunotherapy response. We evaluated the correlation between GINS2 and 6 immune checkpoints.  Figure 8(e) showed that GINS2 was positively correlated with TNFSF9 (*P*  <  0.05), but negatively correlated with TIGIT, IL10, IDO2, CTLA4 and CD80 (*P*  <  0.05). The immune cell infiltration was significantly different in the two groups, with more T cells, follicular helper cells, macrophages M1, and neutrophils in the low GINS2 group (Figure 8(f), *P*  <  0.05). These data together suggest that GINS2 plays an important role in tumor prognosis and may be an immunotherapy response prediction in osteosarcoma.

## 4. Discussion

Osteosarcoma (OS), which occurs frequently in adolescents and adults, is a primary malignant bone tumor [6]. Although the prognosis in patients with OS has improved due to effective therapeutic strategies, patients with distant metastasis or local recurrence still pose an extremely difficult treatment challenge [14]. Therefore, a better understanding of the molecular biology of OS is required in order to improve therapeutic efficiency.

As an important subunit of the GINS complex, GINS2 can mediate the initiation of DNA replication in eukaryotic cells [15]. Relevant research reports pointed out that high expression of GINS2 had a clear connection with the occurrence and development of many malignant tumors, and it could also play an effective role in the tumorigenesis stage by regulating tumor cell apoptosis, signaling pathways, and the cell cycle [16, 17]. However, the role of GINS2 in OS remains unclear to date. Studies have found that GINS2 played a central role as an oncogene in the development stage of OS. When we compared OS with normal bone tissues, we found that the expression of GINS2 in OS samples was up-regulated, which was related to a poor prognosis. Through a series of *in vitro* studies using three OS cell lines, we have demonstrated the core role of GINS2 in cell proliferation and apoptosis. Then, nude mice were injected subcutaneously with HOS cells to study tumor growth *in vivo* in a xenograft model. This experiment verified the *in vitro* results, showing that there was a positive correlation between GINS2 expression and OS growth. In general, our study results revealed sufficient evidence that GINS2 is a prognostic marker and a potential new therapeutic target in OS.

Previous studies reported that GINS2 promoted tumor growth by regulating specific downstream signaling pathways [13, 18]. The regulation of GINS2 by the downstream signaling pathways in tumor cells is not yet fully understood. To examine the potential mechanism by which GINS2 regulated the progression of OS, we performed microarray analysis to investigate the differences in cancer-related genes between ordinary OS cells and GINS2*-*depleted cells. IPA and WB data indicated that GINS2 knockdown cells downregulated the expression of MYC and multiple target genes in the MYC axis. MYC is a proto-oncogene that can improve the performance of oncogenic transcription amplification, is one of the most highly amplified oncogenes in numerous human cancers, and is a significant target of cancer therapy [19–21]. In addition, misregulated expression of MYC is constantly associated with OS oncogenesis and progression [22–24]. In summary, GINS2 might exert an accelerating effect on the activity of the MYC signaling pathway and thus impact OS tumorigenesis and development.

The MYC signal transduction pathway includes the corresponding gene family related to cell proliferation. It is still not clear whether GINS2 is directly or indirectly regulated by the downstream MYC signaling pathway in OS. By performing CoIP-MS analysis and rescue experiments, we obtained potential evidence that the interplay between STAT3 and GINS2 was associated with OS growth. As a transcription factor, STAT3 directly regulates the expression of oncogenes, thereby triggering tumor development [25–27]. Several previous reports have addressed its role as a potential therapeutic target for cancer treatment [28]. A variety of STAT3 inhibitors have also been successfully discovered [29]. In addition, various studies have shown that STAT3/MYC signaling can regulate tumor energy metabolism and the microenvironment to impact tumor cell proliferation and metastasis [30, 31]. These studies have enriched our understanding of how STAT3 can exert an effect on cell proliferation in OS. Therefore, we surmised that GINS2 might regulate the MYC signaling pathway via possible interactions with STAT3 during the carcinogenesis of OS, and utilization of GINS2-STAT3-MYC interaction axis inhibitors might be an effective approach in OS therapy. However, the exact mechanism of MYC pathway regulation by GINS2-STAT3 interaction requires further research. Meanwhile, the mechanism of GINS2 as an immunotherapy response predictor also requires further research in follow-up studies.

## 5. Conclusions

In summary, we have found that high expression of GINS2 is significantly associated with OS tumorigenesis. Furthermore, GINS2 was involved in the regulation of the STAT3/MYC signaling pathway to trigger the growth of OS cells. From a bioinformatics study, GINS2 may be an immunotherapy response prediction in osteosarcoma. Therefore, our findings suggest that GINS2 possesses great potential strategies for treating OS.

## Figures and Tables

**Table 1:** Characteristics of 59 osteosarcoma patients.

	GINS2-positive	GINS2-negative
Gender		
Male	25	15
Female	13	6
Age		
>18 years	21	13
<18 years	17	8
Histotype of tumor		
Conventional	34	19
Other	4	2
Initial tumor site		
Femur	28	17
Tibia	4	2
Humerus	3	1
Fibula	1	1
Rib	1	0
Jaw	1	0
Method of surgery		
Amputation	11	5
Limb salvage	27	16

All factors were well balanced between the two groups (*P*  >  0.05).

## Data Availability

The underlying data supporting the results of this study are available from the corresponding author upon reasonable request.

## References

[1] Zheng D., Liu W., Xie W. (2021). AHA1 upregulates IDH1 and metabolic activity to promote growth and metastasis and predicts prognosis in osteosarcoma. *Signal Transduction and Targeted Therapy*.

[2] Piperno-Neumann S., Le Deley M. C., Redini F. (2016). Zoledronate in combination with chemotherapy and surgery to treat osteosarcoma (OS2006): a randomised, multicentre, open-label, phase 3 trial. *The Lancet Oncology*.

[3] Zhang C., Zheng J. H., Lin Z. H. (2020). Profiles of immune cell infiltration and immune-related genes in the tumor microenvironment of osteosarcoma. *Aging (Albany NY)*.

[4] Ye G., Huang M., Li Y. (2022). The FAP alpha -activated prodrug Z-GP-DAVLBH inhibits the growth and pulmonary metastasis of osteosarcoma cells by suppressing the AXL pathway. *Acta Pharmaceutica Sinica B*.

[5] Yang S., Wang B., Liu C. (2021). THAP9-AS1 promotes tumorigenesis and reduces ROS generation through the JAK2/STAT3 signaling pathway by increasing SOCS3 promoter methylation in osteosarcoma. *Oxidative Medicine and Cellular Longevity*.

[6] Qin Q., Gomez-Salazar M., Tower R. J. (2022). NELL-1 regulates the matrisome to promote osteosarcoma progression. *Cancer Research*.

[7] Kamada K., Kubota Y., Arata T., Shindo Y., Hanaoka F. (2007). Structure of the human GINS complex and its assembly and functional interface in replication initiation. *Nature Structural & Molecular Biology*.

[8] Choi J. M., Lim H. S., Kim J. J., Song O. K., Cho Y. (2007). Crystal structure of the human GINS complex. *Genes & Development*.

[9] Peng L., Song Z., Chen D. (2016). GINS2 regulates matrix metallopeptidase 9 expression and cancer stem cell property in human triple negative Breast cancer. *Biomedicine & Pharmacotherapy*.

[10] Zhang X., Zhong L., Liu B. Z., Gao Y. J., Gao Y. M., Hu X. X. (2013). Effect of GINS2 on proliferation and apoptosis in leukemic cell line. *International Journal of Medical Sciences*.

[11] Gao Y., Wang S., Liu B., Zhong L. (2013). Roles of GINS2 in K562 human chronic myelogenous leukemia and NB4 acute promyelocytic leukemia cells. *International Journal of Molecular Medicine*.

[12] Sun D., Zong Y., Cheng J., Li Z., Xing L., Yu J. (2021). GINS2 attenuates the development of lung cancer by inhibiting the STAT signaling pathway. *Journal of Cancer*.

[13] Liu X., Sun L., Zhang S., Zhang S., Li W. (2020). GINS2 facilitates epithelial-to-mesenchymal transition in non-small-cell lung cancer through modulating PI3K/Akt and MEK/ERK signaling. *Journal of Cellular Physiology*.

[14] Isakoff M. S., Bielack S. S., Meltzer P., Gorlick R. (2015). Osteosarcoma: current treatment and a collaborative pathway to success. *Journal of Clinical Oncology*.

[15] MacNeill S. A. (2010). Structure and function of the GINS complex, a key component of the eukaryotic replisome. *Biochemical Journal*.

[16] Kamada K. (2012). The GINS complex: structure and function. *Subcellular Biochemistry*.

[17] Tian W., Yang X., Yang H., Zhou B. (2020). GINS2 functions as a key gene in lung adenocarcinoma by WGCNA Co-expression network analysis. *OncoTargets and Therapy*.

[18] Huang L., Chen S., Fan H., Ji D., Chen C., Sheng W. (2021). GINS2 promotes EMT in pancreatic cancer via specifically stimulating ERK/MAPK signaling. *Cancer Gene Therapy*.

[19] Dang C. V. (2012). MYC on the path to cancer. *Cell*.

[20] Allen-Petersen B. L., Sears R. C. (2019). Mission possible: advances in MYC therapeutic targeting in cancer. *BioDrugs*.

[21] Beltran H. (2014). The N-myc oncogene: maximizing its targets, regulation, and therapeutic potential. *Molecular Cancer Research*.

[22] Chen D., Zhao Z., Huang Z. (2018). Super enhancer inhibitors suppress MYC driven transcriptional amplification and tumor progression in osteosarcoma. *Bone Res*.

[23] Feng W., Dean D. C., Hornicek F. J. (2020). Myc is a prognostic biomarker and potential therapeutic target in osteosarcoma. *Ther Adv Med Oncol*.

[24] Han G., Wang Y., Bi W. (2012). C-Myc overexpression promotes osteosarcoma cell invasion via activation of MEK-ERK pathway. *Oncology Research Featuring Preclinical and Clinical Cancer Therapeutics*.

[25] Wang Y., Shen Y., Wang S., Shen Q., Zhou X. (2018). The role of STAT3 in leading the crosstalk between human cancers and the immune system. *Cancer Letters*.

[26] Wake M. S., Watson C. J. (2015). STAT3 the oncogene - still eluding therapy?. *FEBS Journal*.

[27] Yu H., Pardoll D., Jove R. (2009). STATs in cancer inflammation and immunity: a leading role for STAT3. *Nature Reviews Cancer*.

[28] Yang L., Lin S., Xu L., Lin J., Zhao C., Huang X. (2019). Novel activators and small-molecule inhibitors of STAT3 in cancer. *Cytokine & Growth Factor Reviews*.

[29] Huang Q., Zhong Y., Dong H. (2020). Revisiting signal transducer and activator of transcription 3 (STAT3) as an anticancer target and its inhibitor discovery: where are we and where should we go?. *European Journal of Medicinal Chemistry*.

[30] Gao S., Chen M., Wei W. (2018). Crosstalk of mTOR/PKM2 and STAT3/c-Myc signaling pathways regulate the energy metabolism and acidic microenvironment of gastric cancer. *Journal of Cellular Biochemistry*.

[31] Nowak D. G., Cho H., Herzka T. (2015). MYC drives pten/trp53-deficient proliferation and metastasis due to IL6 secretion and AKT suppression via PHLPP2. *Cancer Discovery*.

